# Supplementary material for: Elucidation of Pharmacological Mechanism Underlying the Anti-Alzheimer’s Disease Effects of Evodia rutaecarpa and Discovery of Novel Lead Molecules: An In Silico Study
Source: Molecules. 2023 Aug 3;28(15):5846. doi: 10.3390/molecules28155846 (PMC10421504; doi:10.3390/molecules28155846)
Supplement: Supplementary file 1 [file molecules-28-05846-s001.zip › Table S2 Toxicity prediction of 32 active compounds of WZY.pdf]

Table S2 Toxicity prediction of 32 active compounds of WZY

Code	Compound name	Predicted LD50 (mg/kg)	Toxicity Class	Hepatotox-icity	Carcinog-enicity	Immunot-oxicity	Mutage-nicity	Cytoto-xicity
WZY1	1-methyl-2-nonyl-4-quinolone	200	3	inactive	inactive	active	active	inactive
WZY2	1-methyl-2-undecyl-4-quinolone	200	3	inactive	inactive	active	active	inactive
WZY23	1-Methyl-2-[(Z)-5-Undecenyl]-4(1H)-Quinolone	200	3	inactive	inactive	active	active	inactive
WZY28	Echinopsine	347	4	inactive	inactive	inactive	active	active
WZY3	Evocarpine	200	3	inactive	inactive	active	active	inactive
WZY4	1-methyl-2-[(Z)-undec-6-enyl]-4-quinolone	200	3	inactive	inactive	active	active	inactive
WZY5	1-methyl-2-[(Z)-pentadec-10-enyl]-4-quinolone	200	3	inactive	inactive	active	active	inactive
WZY7	1-methyl-2-pentadecyl-4-quinolone	200	3	inactive	inactive	active	active	inactive
WZY11	Evodiamine	780	4	inactive	inactive	inactive	active	inactive
WZY12	icosa-11,14,17-trienoic acid methyl ester	1500	4	inactive	inactive	inactive	active	inactive
WZY13	N-(2-Methylaminobenzoyl) tryptamine	1500	4	inactive	inactive	inactive	inactive	inactive
WZY16	rutaecarpine	1400	4	inactive	inactive	inactive	active	inactive
WZY17	dihydrorutaecarpine	780	4	inactive	inactive	inactive	active	inactive
WZY18	Goshuyuamide I	650	4	inactive	inactive	inactive	active	inactive
WZY21	GoshuyuamideII	1400	4	inactive	inactive	inactive	inactive	inactive
WZY26	N, N-Dimethyl-5-Methoxy Tryptamine	963	4	inactive	inactive	active	inactive	inactive
WZY27	Dehydroevodiamine	2000	4	inactive	inactive	active	active	inactive
WZY30	14-Formyldihydrorutaecarpine	780	4	inactive	inactive	inactive	active	inactive
WZY32	1,2,3,4-Tetrahydro-1-Oxo-Î’-Carboline	1500	4	inactive	inactive	inactive	inactive	inactive
WZY20	hydroxyevodiamine	780	4	inactive	inactive	active	active	inactive
WZY29	Rhetsinine	1500	4	inactive	inactive	inactive	active	inactive
WZY15	1-(5,7,8-trimethoxy-2,2-dimethylchromen-6-yl) ethanone	500	4	inactive	active	active	inactive	inactive
WZY19	berberine	200	3	inactive	active	active	active	active
WZY22	2-Hydroxy-3-formyl-7-methoxycarbazole	1200	4	active	inactive	active	active	inactive
WZY25	Graveoline	400	4	inactive	active	inactive	active	inactive
WZY31	Kokusaginine	1000	4	inactive	active	active	active	inactive
WZY14	Fordimine	220	3	inactive	inactive	inactive	inactive	inactive
WZY24	Goshuyic Acid	10000	6	inactive	inactive	inactive	inactive	inactive
WZY6	icosa-11,14,17-trienoic acid methyl ester	20000	6	inactive	inactive	inactive	inactive	inactive
WZY10	sitosterol	890	4	inactive	inactive	active	inactive	inactive
WZY8	24-methyl-31-norlanost-9(11)-enol	2000	4	inactive	inactive	active	inactive	inactive
WZY9	beta-sitosterol	890	4	inactive	inactive	active	inactive	inactive

* Class I: fatal if swallowed (LD50 ≤ 5), Class II: fatal if swallowed (5 < LD50 ≤ 50), Class III: toxic if swallowed (50 < LD50 ≤ 300), Class IV: harmful if swallowed (300 < LD50 ≤ 2000), Class V: may be harmful if swallowed (2000 < LD50 ≤ 5000), Class VI: non-toxic (LD50 > 5000).
